# Crowdsourcing care: What general practitioner WhatsApp groups get right … and wrong

**DOI:** 10.4102/safp.v68i1.6325

**Published:** 2026-06-29

**Authors:** Magrietha Swart

**Affiliations:** 1 Dr AP Swart Inc., Molteno Anaesthetics, Cape Town, South Africa

**Keywords:** *POPI Act*, peer support, WhatsApp group, data breach, referral, privacy, medical data

## Abstract

With the growing use of WhatsApp groups by general practitioners, there are clear pros and cons to their use. Peer support, easing referral and educational opportunities are thereby supported, but at the cost of potential privacy and data protection issues because of the medium and nature of the technology, and potential legal ramifications of the *Protection of Personal Information Act 4 of 2013*.

**Contribution:**

The use of such groups requires care and attention, and although especially useful, securing data and confidentiality is a potential cause of concern.

## Introduction

Since the explosion of telemedicine during the coronavirus disease 2019 (COVID-19) pandemic,^1^ many medical practitioners, especially general practitioners (GPs), have moved to asking clinical-based questions in private medical social media groups. In the last 2 years, GP WhatsApp groups like *GP chats* have brimmed with thousands of doctors asking each other’s advice, from medical aids to clinical questions surrounding patients in their care. This is happening not only in South Africa but worldwide, as the availability of smartphones and various applications eases communication and information gathering, though this is not without risk.^2^

## Rationale

But why do these groups exist? The overarching theme seems to be that there is a patient demand on GPs to investigate conditions and do extensive workups that might not be within the natural scope of a GP. This results in an expectation of almost limitless knowledge that the GP now must have in the age where patients are using artificial intelligence (AI) and the Internet search engine, Google, to double and triple check every diagnosis, our competition as GPs is steep. More educated patients report much lower positive experiences with primary healthcare^3^, and with the frequent use of internet sources and rife miscommunication on health issues, this significantly impacts the GP’s ability to provide quality care in a timeous fashion.^4^

Most of the questions posed by the GPs in these groups are usually followed by their suspected diagnosis, and many times the diagnosis is correct, but the GP remains unsure enough to pose the question in the first place. Contributing factors may be a very real time crunch in primary care, where the GP is expected to solve everything in a 15 min window, and this can be daunting, especially for new practitioners. *The buck stops with me* can be overwhelming and isolating, and as most GPs do not readily have access to specialists, their next best place to turn would be their colleagues who might have had experience in the problem they are facing.

How do these groups help? In some instances, with regard to referral protocols, contact numbers for specialists, locum opportunities and medical aid claims advice, these groups are very helpful. The clinical cases that the doctors posted are also posted under the agreement that the patient has consented to it and the peer reassurance the GP receives from their colleagues helps. Many GPs have different fields of interest, and the wide scope of knowledge provided is priceless. There are also a few qualified specialists in the group and registrars in specific fields who provide advice. Additionally, when a patient warrants a referral to a specialist, peer guidance will often be helpful for the GP to decide that this patient would be best managed by a specialist in a specific field. The observing GPs also have chances to see cases that they would not always see. Sometimes, weeks after the GP group receives feedback, it helps and supports future diagnostic queries. I specifically remember seeing cases of Madura foot and Mango fly infestation on GP groups that I have never seen in private practice in Cape Town. On a personal level, some GPs have also confessed feeling overwhelmed, scared or unsure in the group and have received a huge outpouring of support.

## Limitations of the medium

Can we do this? Unfortunately, nothing in medicine, even a WhatsApp support group, is ever simple. The first enormous issue that comes in is patient privacy. The advent of the biggest GP WhatsApp group in South Africa came from a link from a medical doctors’ Facebook group created a decade ago with over 15 000 members. Although there are screening questions to join this Facebook group, there are medical supply companies and locum recruiters on this group as well, and it would be easy for a non-doctor to join this Facebook group, then have a direct link to the GP WhatsApp groups. The WhatsApp groups have rules, but there is no area in these groups where practice numbers or registration is supplied to the admin to check that the members are all medical doctors. This obviously raises a huge red flag, because even if you have consent from a patient to anonymously post photos of their condition or results to your 1000 colleagues in the group, how can you confirm that every eye in there is a colleague?

A retrospective analysis of WhatsApp communication at a district South African hospital found that 3.3% of messages contained non-anonymised patient information, illustrating that privacy breaches are a real concern. A further 3.3% of messages contained patient information that had been anonymised, so some attempt had been made to minimise this risk, but such quick communication sometimes falls short in an effort to rapidly get an answer.^5^

Another core issue is that the information the GP posts will only be what they deem relevant, which may already be biased towards a diagnosis and therefore lead. There may be more image-based diagnoses versus exam-based diagnoses made, which in the long term could lead to more groupthink and the inclination to prefer speed to diagnosis rather than reflection on the patient in front of you. There are strategies for minimising diagnostic error,^6^ but controlling for your own bias is a difficult thing to do in the context of medical consensus, and such posting may lead to framing bias with a possible false consensus, as not every member will be responding, but may give the impression that everyone is agreeing to it.^7^

As health information is classified as special personal information under the *Protection of Personal Information Act 4 of 2013* (*POPIA*) with a high threshold of protection, it needs to be dealt with a great deal of care.^8^ Whilst there is provision for clinical information being shared between acting clinicians, even those acting clinicians are not allowed to store medical records on their phones. The use of these WhatsApp groups raises concern for meaningful anonymisation (which does not only include withholding of names and ID number, but also identifiable factors like tattoos or even rare medical conditions), data protection and the suitability of WhatsApp as a platform, as WhatsApp tends to automatically backup data to the cloud which might include these WhatsApp images and information to all the users in the groups clouds.

## Conclusion

At the end of the day, the diagnosis, patient consent, and data protection remain the treating GP’s duty and a shared diagnosis does not mean a shared responsibility.

Moving forward with these WhatsApp groups would need to change. Although peer-to-peer relationships are extremely important in medicine, and this cannot be stated enough, patient confidentiality and protection must come first. Currently, the risk of data leak outweighs the benefit of a GP discussion based around clinical cases.
